# UNDIFFERENTIATED PLEOMORPHIC SARCOMA: PROGNOSTIC FACTORS IN 42 EXTREMITY CASES

**DOI:** 10.1590/1413-785220233102e265942

**Published:** 2023-05-01

**Authors:** CARLOS HENRIQUE MAÇANEIRO, ANDRÉ MATHIAS BAPTISTA, OLAVO PIRES DE CAMARGO, RENÉE ZON FILIPPI, EVANDRO TITO OLIVEIRA

**Affiliations:** 1 Universidade de Sao Paulo, Faculdade de Medicina, Hospital das Clínicas, Instituto de Ortopedia e Traumatologia, Sao Paulo, SP, Brazil.

**Keywords:** Histiocytoma, Malignant Fibrous. Sarcoma. Prognosis. Recurrence. Death. Survival, Histiocitoma Fibroso Maligno, Sarcoma, Prognóstico, Recidiva, Morte, Sobrevida

## Abstract

**Introduction:**

Soft tissue undifferentiated pleomorphic sarcoma (UPS) in extremities is considered a rare neoplasm, corresponding to 5% of soft tissue sarcomas (STS) today. The objective was to evaluate prognostic factors related to death, local recurrence (LR), and impact on survival rates.

**Methods:**

A retrospective study including 42 patients with UPS in extremities treated surgically in a single center. Comparisons were made between demographic data, characteristics of the neoplasia, and treatment. Between the variables with statistical significance, logistic regression analysis was used. Survival rates were evaluated using Kaplan-Meier plots. To compare the effect of variables on survival rates, the Log-Rank test was used.

**Results:**

Age group of patients was from 25 to 85 years (mean 58 years), with a mean follow-up of 29.6 months. The variables with the highest effect on survival rates were sizes larger than 15 cm (T4) with p = 0.01, presence of metastatic lesions, and prognostic stage IV according to the American Joint Committee of Cancer (AJCC) with p < 0.001. The mean survival was 25.9 months. Metastasis and stage IV of AJCC were associated with a reduction in patient survival (17.8 months) with Log-Rank test p < 0.001. **Conclusion:** The main factors of poor prognosis related to mortality and reduction of survival of UPS in extremities were metastatic lesions and stage IV of AJCC. ***Level of Evidence III, Retrospective Study.***

## RESUMO

O sarcoma pleomórfico indiferenciado (SPI) de tecidos moles em extremidades, neoplasia rara, correspondente a 5% dos sarcomas de tecidos moles (STM).

**Objetivo:**

Avaliar fatores de prognóstico relacionados a óbito, recorrência local (RL) e impacto na sobrevida geral.

**Métodos:**

Estudo retrospectivo de 42 pacientes com SPI tratados cirurgicamente em centro único. Foram comparados dados demográficos, características da neoplasia e de tratamento; e para as variáveis com significância estatística, foi realizada análise de regressão logística. A sobrevida foi avaliada através de gráficos de Kaplan-Meier; e os efeito das variáveis sobre a sobrevida, por meio do teste de log-rank.

**Resultados:**

Os pacientes tinham idades entre 25 e 85 anos (média de 58 anos), com seguimento ambulatorial médio de 29,6 meses. As variáveis com maior relação com o desfecho óbito foram tamanho maior que 15 cm (T4), com p = 0,01, metástases e estágio IV de prognóstico da American Joint Committee on Cancer (AJCC), com p < 0,001. A sobrevida média dos pacientes foi de 25,9 meses. Presença de metástase e estágio IV da AJCC foram associados à redução na sobrevida dos pacientes (17,8 meses; p < 0,001). **Conclusão:** Os principais fatores de mau prognóstico relacionados ao óbito e à redução da sobrevida dos pacientes com SPI foram doença metastática e estágio IV da AJCC. **Nível de Evidência III, Estudo Retrospectivo.**

## INTRODUCTION

Undifferentiated pleomorphic sarcoma (UPS) of soft tissues was initially described in the 1960s in case reports developed by Kaufman and Stout^1^ and Ozzello, Stout, and Murray. ^(^ ^2^ In the 1970s, Weiss and Enzinger^3^ conducted an extensive analysis of cases, describing the neoplasm in detail and establishing it as a primitive mesenchymal lesion with partially fibroblastic and histiocytic differentiation. UPS usually presents in men over 50 years old, affecting the lower limbs in most cases. Due to its deep structure origin, there is difficulty in early diagnosis and management, with approximately 5% of cases diagnosed with metastatic lesions, the lungs being the main site. ^(^ ^4^ ^), (^ ^5^

Identifying prognostic factors is one of the main approaches used to improve the efficacy of cancer treatments. These factors are important both in practice and in conducting trials and clinical studies. On the one hand, to select and plan the most appropriate treatment, and on the other hand, to enable the analysis of differences in possible study outcomes and identify subgroups for new treatment possibilities. ^(^ ^6^

Due to the various phases related to the evolution of UPS diagnosis since 1960, much of the published data regarding prognostic factors are still associated with the period prior to 2002 when it was still considered as Malignant Fibrous Histiocytoma (MFH) and accounted for virtually 60-70% of soft tissue sarcoma (STS) diagnoses, making the application of these factors inadequate, when considering only UPS cases. ^(^ ^7^

Given the lack of studies available in the Brazilian literature and elaborated by orthopedists involving prognostic factors of UPS, this research focuses on conducting a retrospective analysis of these factors over a period of 30 years. Therefore, the objectives of the study were to identify possible prognostic factors related to death or local recurrence and to identify possible factors that may have an impact on the survival rates of these patients.

## METHODS

A retrospective study, conducted at a single center (Institute of Orthopedics and Traumatology of the Hospital das Clínicas of the Universidade de São Paulo [IOT HC FMUSP]) from January 1988 to December 2018. The study was approved by the local ethics committee (CAAE 80667617.2.0000.0068).

### Patient-related factors

A total of 202 cases of patients with the diagnosis of MFH or UPS were selected from the institution. All cases were evaluated and classified by two pathologists from the institute, both specialists in musculoskeletal sarcoma cases. Cases considered inoperable (1), located in the head and/or neck, chest, trunk, bones (2), and with any type of cellular differentiation reported in the postoperative pathological report (3) were excluded. Thus, 42 patients were included in the study.

### Treatment-related factors

All patients underwent surgical treatment at the institution, with wide and/or radical resection. Surgical margins were classified as negative (the narrow margin was considered negative) or positive, according to the Enneking’s stages. ^(^ ^8^ Cases requiring surgical reapproach (debridement, margin enlargement, amputations, and disarticulations) and those who received any type of clinical treatment (radiotherapy or chemotherapy) were also listed.

### Variables and outcome measures

All included UPS were considered to be of high histological grade according to the criteria of the French Federation of Cancer Centers (FNCLCC).^9^ Follow-up time was measured in months, within the period between the first and last consultation recorded in the medical records. Lesion size was in accordance with the pathological report, (greater axis in cm macroscopically) and classified according to the soft tissue sarcoma classification from the American Joint Committee on Cancer (AJCC - T1 up to 5 cm, T2 from 6 to 10 cm, T3 from 11 up to 15 cm, and T4 greater than 15 cm). ^(^ ^10^ Local recurrence (LR) and distant metastases or lymph node involvement were also reported. Survival rates were estimated from the date of surgical treatment until the date of death or last consultation recorded in the medical records or online system.

### Statistical analysis

The data were analyzed using R software version 4.2.0. Continuous variables were described using mean, median, standard deviation, interquartile range, minimum and maximum values. Categorical variables were described using counts and percentages. The normal distribution of continuous variables was measured using the Shapiro-Francia test. For comparison of continuous variables between two groups, either the T-test or the Wilcox test was used depending on the distribution of the variable. Categorical variables were analyzed using Fisher’s exact test for two groups, whereas the chi-square test was used for three or more groups. In addition, logistic regression was used to identify the effect of candidate variables on death or local recurrence. Finally, actuarial tables and Kaplan-Meier graphs were used for survival rates analysis of the patients, and the Log-rank test was used to compare the effect of a variable on patient survival. P values lower than 0.05 were considered significant.

## RESULTS

Table 1 presents clinical and demographic characteristics of patients.

**Table 1:** Demographic, pathological, and treatment characteristics of 42 patients with UPS.

Variables	Total (%)
Gender	
Men	(20) 47
Women	(22) 53
Age	
Mean	58
Standard deviation	± 14
Age group	25-85
Site	
Upper limb	12 (29)
Lower limb	(30) 71
Laterality	
Left	(30) 71
Right	12 (29)
Follow-up (months)	
Medium	29.6
Minimum	0.6
Maximum	165.9
Size (cm)	
T1 (0-5)	1 (2)
T2 (6-10)	10 (24)
T3 (11-15)	10 (24)
T4 (> 15)	21 (50)
Size (cm)	
Mean	9
Variation	2-23
Surgery	
Wide resection	(30) 71
Amputation/disarticulation	12 (29)
Margin	
Marginal or intralesional	37 (88)
Affected	5 (12)
Radiotherapy	
Yes	26 (62)
Preoperative	10 (38)
Postoperative	16 (62)
No	16 (38)
Chemotherapy	
Yes	25 (59)
Neoadjuvant	10 (40)
Adjuvant	15 (60)
No	17 (41)
Local recurrence	
Yes	6 (14)
No	36 (86)
Resurgery (two or more surgical procedures)	
Yes	9 (22)
No	33 (78)

### Deaths

A total of 31 patients died during the study period (74%). The variables that showed some relation with a higher chance of death outcome were: having a size larger than 15 cm (T4) (p = 0.01), having stage IV of AJCC prognosis (p < 0.001), and having metastasis (p < 0.001) (Table 2).

**Table 2:** Variables with statistical significance regarding the occurrence of death.

Variable	Items	Death		Total	Test
		Yes	No		
**Stage of injury**	IIIA	0 (0%)	5 (100%)	5 (11.90%)	P < 0.0001 (Pearson's Chi-Square Test)
	IIIB	3 (50%)	3 (50%)	6 (14.29%)	
	IV	28 (90.32%)	3 (9.68%)	31 (73.81%)	
	**Total**	31 (73.81%)	11 (26.19%)	42 (100%)	
**Size of injury**	T1-T3	12 (57.14%)	9 (42.86%)	21 (50%)	P = 0.0140 (Fisher's Exact Test)
	T4	19 (90.48%)	2 (9.52%)	21 (50%)	
	**Total**	31 (73.81%)	11 (26.19%)	42 (100%)	
**Presence of metastasis**	No	3 (27.27%)	8 (72.73%)	11 (26.19%)	P = 0.002 (Fisher's Exact Test)
	Yes	28 (90.32%)	3 (9.68%)	31 (73.81%)	
	**Total**	31 (73.81%)	11 (26.19%)	42 (100%)	

To identify which factors have a greater effect on the risk of death, a logistic regression was performed, which showed that the presence of metastasis and the stage of the lesion were related to an increased risk of death in patients (Table 3). Note that due to the perfect correlation between the presence of metastasis and the occurrence of grade IV tumors, two models were made to demonstrate that both variables had identical coefficients.

**Table 3:** Logistic regression models regarding the occurrence of death and their respective coefficients.

	Metastasis	Tumor grade
(Intercept)	-1.74	-1.74
	-1.08	-1.08
Gender (men)	0.23	0.23
	-1.01	-1.01
Metastasis (yes)	3.14*	N/A
	**-1.01**	
Lesion size (T4)	1.74	1.74
	-1.05	-1.05
Stage of injury (IV)	N/A	3.14*
		**-1.01**
N	42	42
Pseudo R2	0.53	0.53

*p < 0.01; ( ): standard deviation.

### Local recurrence

Six patients presented tumor recurrence (14%). None of the studied variables presented statistical significance with the recurrence outcome, however male patients showed a higher tendency to LR (Table 4).

**Table 4:** Demographic data of the population in relation to the occurrence of local recurrence of the lesion.

Variable	Items	Local Recurrence		Total	Test
		Yes	No		
**Older than 60 years**	No	3 (14.29%)	18 (85.71%)	21 (50.00%)	p = 1.0000 (Fisher's Exact Test)
	Yes	3 (14.29%)	18 (85.71%)	21 (50.00%)	
	**Total**	6 (14.29%)	36 (85.71%)	42 (100.00%)	
**Age**	Min/Max	36.0/74.0	25.0/85.0	25.0/85.0	p = 0.7322 (T test)
	Med [IQR]	62.5 [56.5;69.2]	59.5 (-51.2;67.5)	59.5 [52.0;69.2]	
	Average (SD)	60.2 (13.7)	57.9 (15.0)	58.2 (14.6)	
	**N**	6 (14.29%)	36 (85.71%)	42 (100.00%)	
**Gender**	Men	5 (25.00%)	15 (75.00%)	20 (47.62%)	p = 0.0866 (Fisher's Exact Test)
	Women	1 (4.55%)	21 (95.45%)	22 (52.38%)	
	**Total**	6 (14.29%)	36 (85.71%)	42 (100.00%)	

Med: median; IQR: interquartile range; SD: standard deviation.

### General survival rates

**Figure 1:**
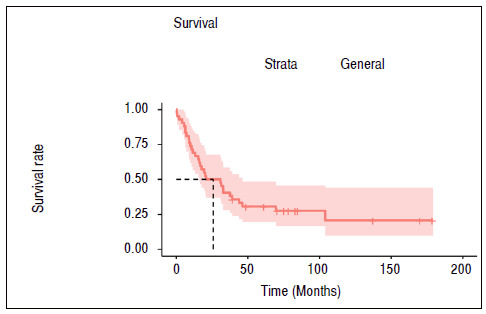
Survival time without death of the cohort studied. Dotted line indicates median survival time of the population.

In addition, patient survival rates were demonstrated using a Kaplan-Meier chart, in which it was found that the mean survival rate of patients was 25.9 months (Table 5 and Figure 1).

**Table 5:** Table containing the results of survival tests for death.

Item	Time up to 50% survival	LOGRANK Test
General	25 months	--
Presence of metastasis	Yes: 17.8; No: N/A	p < 0.001
Stage of injury	IIIA-B: N/A; IV: 17.8	p < 0.001

N/A: not applicable (population did not reach values less than 50% survival).

**Figure 2:**
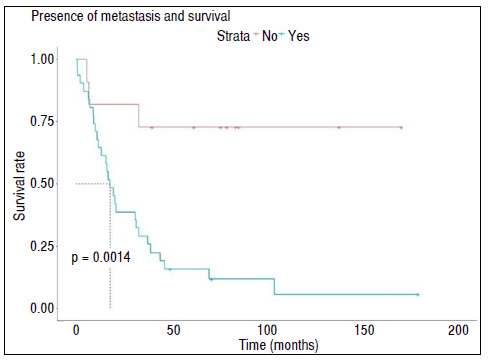
Survival time without death of the cohort studied in relation to the presence of metastasis. Dotted line indicates median survival time of the subpopulation. Dotted line indicates median survival time of the subpopulation.

**Figure 3:**
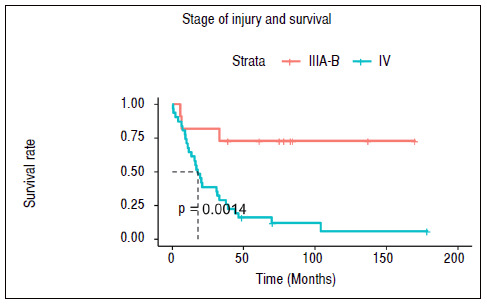
Survival time without death of the cohort studied in relation to the stage of the lesion. Dotted line indicates median survival time of the subpopulation.

Finally, we observed the presence of metastasis or of stage IV lesion were significantly associated with a reduction in patient survival rate (Table 5 and Figures 2 and 3).

## DISCUSSION

**Figure 4:**
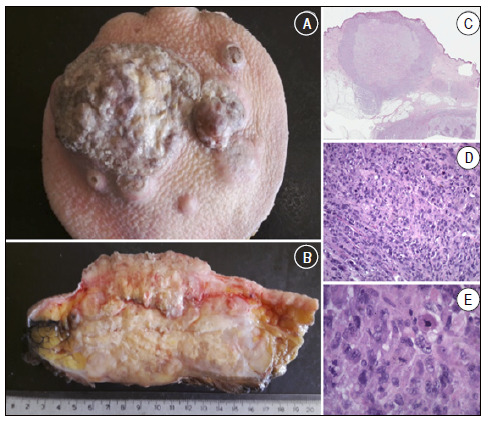
A and B: Macroscopy; soft tissue tumor in the hip, surface with ulcerated center. Affects a deep margin; C, D and E: Microscopy (H&E) revealing cytological and nuclear pleomorphism. The tumors often contain giant cells, spindle cells, and histiocyte-like round cells in varying proportions. Storiform cell patterns and chronic inflammatory stroma are also common. The spindle component resembles fibroblasts, myofibroblasts, or smooth muscle patterns. ^(^ ^4^

Source: A cortesy from pathology IOT HC FMUSP/CEDAP Joinville.

Aggressive clinical behavior is the only aspect of MFH/UPS that remained unchanged since its initial descriptions by Kauffman and Stout; ^(^ ^1^ Ozello, Stout, and Murray; ^(^ ^2^ and Kempson and Kyriakos. ^(^ ^11^ This pathology usually presents with high-grade histological lesions, deep location, and the capacity to reach dimensions larger than 10-15 cm. Unfortunately, it also has high rates of metastatic disease and recurrences, which directly influence disease-free survival and overall survival. ^(^ ^3^ ^), (^ ^9^ ^), (^ ^12^ As MFH/UPS was a leading diagnosis for STS during much of the 1990s, it influenced a whole generation of studies (Figure 4). ^(^ ^13^ ^), (^ ^14^

With the evolution of auxiliary techniques within the surgical field, MFH/UPS has been gradually losing relevance and, today, it is considered a diagnosis of exclusion that corresponds to approximately 5% of STS. ^(^ ^4^ Thus, being a relatively “new” diagnosis, our study evaluated the main prognostic factors chronologically to compare and analyze possible divergences.

Of the patients involved in the study, 74% died. Using univariate analysis, the variables that showed some relation with a higher chance of death outcome were having a size larger than 15 cm (T4) (p = 0.01), (p < 0.001), having metastasis (p < 0.001), and having stage IV of AJCC prognosis. Since all stage IV lesions present metastasis, it can be affirmed that they are related variables, a fact that is confirmed through two logistic regression models (Table 3), with identical coefficients. Lesion size and gender showed no relationship after multivariate analysis.

These results are similar to the findings of the “new” concepts about the UPS classification.

Before 2002, when there was a change of nomenclature from MFH to UPS, Markhede, Angervall, and Stener^15^ analyzed the STS in a multivariate way and pointed out that there is an increased risk of death in patients with recurrence of injury (p < 0.01), especially in cases of recurrence in the first two years after surgical treatment. In the post-reclassification phase of MFH, Vodanovich et al. ^(^ ^16^ in a multivariate analysis of 266 UPS defined that each passed year since lesion and metastasis diagnosis increased the death chance (risk ratio 1.03 and 2.89, p < 0.001 and p = 0.001 respectively) and that lesions in the upper limbs have a lower chance of death (risk ratio 0.57 and p value = 0.043).

Around 15% of the sample presented LR. Among the variables analyzed, none presented statistical significance, but men showed a tendency to have LR of the lesions (p = 0.086). Age and stage of injury did not show statistical significance.

Kearney, Soule, and Ivins^12^ evaluated 167 cases of STS retrospectively, with an LR rate of 51% and more than half with more than one episode. Among patients with fascia superficial tumors, 71% presented LR after “complete excision”; high rates are apparently associated with less aggressive resections, longer survival (therefore, more timely for recurrence), and greater ease for detection of palpation. In addition, of the superficial cases, 31% were diagnosed as deep lesions to the fascia. Markhede, Angervall, and Stener, ^(^ ^15^ Pisters et al. ^(^ ^17^ and Gibbs et al. ^(^ ^18^ presented statistically relevant results, relating LR with inadequate surgeries or margin of the affected lesion in up to 76.2% of the relapsed disease in patients.

Considering recent evaluations (after 2002), Vodanovich et al. ^(^ ^16^ and Ozcelik et al. ^(^ ^19^ also showed results associating RL with affected margins in univariate and multivariate analyses, a fact that does not corroborate with the data shown in the present study.

The recurrence variable also correlates with radiotherapy treatment. Ozcelik et al. ^(^ ^19^ reported good results considering perioperative radiotherapy (p = 0.009 and p = 0.000 respectively).

Although it is a variable without impact in our cohort, advanced age showed statistical significance in the prospective study of Pisters et al. ^(^ ^17^ for patients ≥ 50 years. Vodanovich et al. ^(^ ^16^ also presented statistical significance in multivariate analysis for patients ≥ 70 years (p < 0.016, RR 1.6; p = 0.046, HR 1.03).

The authors speculate that such differences may occur due to the small sample size added to the high mortality rate of the disease causing few patients in the sample to present LR.

The mean survival of the patients in this study was demonstrated through the Kaplan-Meier graph (Figure 2), with an average of 25.9 months. Presence of metastasis or stage IV AJCC prognosis presented close relationship with the reduction of survival rates, with a mean of 17.9 months (p < 0.001).

Among the analyses that precede the reclassification of MFH, Kearney, Soule, and Ivins, ^(^ ^12^ Pisters et al., ^(^ ^17^ and Gibbs et al. ^(^ ^18^ demonstrated that proximal tumors that affect the pelvis and proximal region of the thigh, are of worse prognosis, affecting the survival rates of these patients; the last two authors consider them as independent prognostic factors.

The size of tumors was also associated with worse survival by some authors. Pezzi et al. ^(^ ^13^ described that survival rates were 82% in patients with lesions up to 5 cm, 68% in cases of 5-10 cm, and 51% for lesions greater than 10 cm with p < 0.05. Pisters et al. ^(^ ^17^ and Gibbs et al. ^(^ ^18^ corroborate with the finding through multivariate analysis, in which tumors ≥ 10 cm showed a high correlation with worse survival rates (risk ratio 1.5 and P < 0.001, respectively). However, in the studies by Kearney, Soule, and Ivins^12^ and Markhede, Angervall and Stener, ^(^ ^15^ tumor sizes had no impact on survival, a fact that aligns with the results of our study.

The histological grade of tumors was another constant variable in survival analyses, considered a poor prognostic factor. ^(^ ^15^ ^), (^ ^17^ ^), (^ ^18^ Gibbs et al. ^(^ ^18^ and Pisters et al. ^(^ ^17^ also evaluated patients at the time of lesion diagnosis and patients who already presented metastatic lesions or local recurrence to measure their effects on survival rates (p = 0.0002 and risk of 1.5, respectively).

Correlating studies from different periods, the main prognostic factors related to worsening of survival rates were size (p = 0.02) and presence of metastasis at diagnosis (p = 0.001) increasing the risk of mortality by almost 500% compared to patients without metastasis. ^(^ ^17^ The presence of metastatic lesions during the disease showed a negative correlation with survival in our study and in the analyses of Ozcelik et al. ^(^ ^19^ with p < 0.001 and = 0.004, respectively.

### Study limitations

The short time to conduct the project and the rare diagnosis (small sampling) studied requires a retrospective design study. This design brings disadvantages, mainly due to systematic errors. Selection bias occurred since it is a single reference center for cancer cases, so there is a higher incidence of severe cases. Confusion bias can also end up influencing outcomes, such as survival rates. Since not every case has the defined cause of death (related to the disease itself or another secondary cause), comorbidities may end up influencing outcomes. The fact that patients received orthopedic and clinical care in independent sectors also generates limitations since it restricts information, such as the chemotherapy and radiotherapy protocols and the exact date of occurrence or recurrence of metastasis. Immunohistochemical reassessment of each case became financially unfeasible since it is a study with independent funding.

## CONCLUSIONS

This study demonstrated that the main factors of poor prognosis related to death were size larger than 15 cm (larger diameter), metastatic disease, and stage IV of AJCC prognosis; for recurrence, no variable studied presented statistical relevance, although male patients showed a higher tendency to LR. In terms of survival rates, the main factors related were the presence of metastatic lesions and the stage IV of prognosis.

Despite this study being relevant to assist medics in the development of strategies for the treatment of patients, new studies with larger cohorts and with the possibility of immunohistochemical reassessment are necessary to confirm our findings.
